# An Innovative Approach of Parameter Loading Path Design for Grain Refinement and Its Application in Ni80A Superalloy

**DOI:** 10.3390/ma14216703

**Published:** 2021-11-07

**Authors:** Guo-Zheng Quan, Yan-Ze Yu, Xue Sheng, Kun Yang, Wei Xiong

**Affiliations:** 1 Chongqing Key Laboratory of Advanced Mold Intelligent Manufacturing, School of Material Science and Engineering, Chongqing University, Chongqing 400044, China; yyz6201314@sina.com (Y.-Z.Y.); shengxue0305@sina.com (X.S.); yk9999026@sina.com (K.Y.); 2 State Key Laboratory of Materials Processing and Die & Mould Technology, Huazhong University of Science and Technology, Wuhan 430074, China; 3 Collaborative Innovation Center of Advanced Nuclear Energy Technology, Key Laboratory of Advanced Reactor Engineering and Safety of Ministry of Education, Institute of Nuclear and New Energy Technology, Tsinghua University, Beijing 100084, China

**Keywords:** Ni80A superalloy, microstructural evolution mechanisms, grain refinement, parameter loading path design

## Abstract

In order to obtain the desired mechanical properties of products, an innovative method of loading parameter designs for acquiring the desired grain refinement is proposed, and it has been applied in the compression process of Ni80A superalloy. The deformation mechanism maps derived from processing maps based on the Dynamic Materials Model (DMM) theory were constructed, since the critical indicator values corresponding to dynamic recrystallization (DRX) and dynamic recovery (DRV) mechanisms were determined. The processing-parameter domains with DRX mechanisms were separated from the deformation mechanism map, while such domains were chaotic and difficult to apply in innovative parameter loading path design. The speed-loading path derived from strain rate-loading path in a compression process was pursued. The grain refinement domains are discretized into a finite series of sub-domains with clear processing parameters, and the optimal strain rate of each sub-domain is determined by step-by-step finite element simulation. A 3D response surface of the innovative optimal loading path of strain rate was fitted by interpolating methods. Finally, the isothermal compression experiments for Ni80A superalloy were conducted, and the microstructure observations indicated that the desired grain refinement was achieved. This innovative method of parameter loading path design contributes to the microstructure adjustment of the alloys with DRX mechanism.

## 1. Introduction

Ni80A superalloy has a wide application in the hot-end components of prime movers due to the advantages of high strength and excellent corrosion-resistance at elevated temperature [1,2,3]. The hot-end components with high strength and toughness requirements are always manufactured by thermo-plastic deformation processes [4,5]. The grain size with a lower level is considered as a crucial indicator to adjust the comprehensive mechanical properties related with grain boundaries of alloys [6,7]. In a thermo-plastic deformation process three basic microstructural evolution mechanisms, including dynamic recrystallization (DRX), dynamic recovery (DRV), and grain growth (GG), co-exist and compete dynamically [8,9,10,11,12]. The grain size is determined by the combined effect of the grain refinement mechanism, DRX, and the grain coarsening mechanism, GG. Meanwhile, DRV affects grain morphology [13]. Consequently, before the optimized process design for acquiring refined grains, the detailed description of microstructural evolution mechanisms in thermo-plastic deformation is proposed. However, it is a significant and difficult issue.

**Figure 1:**
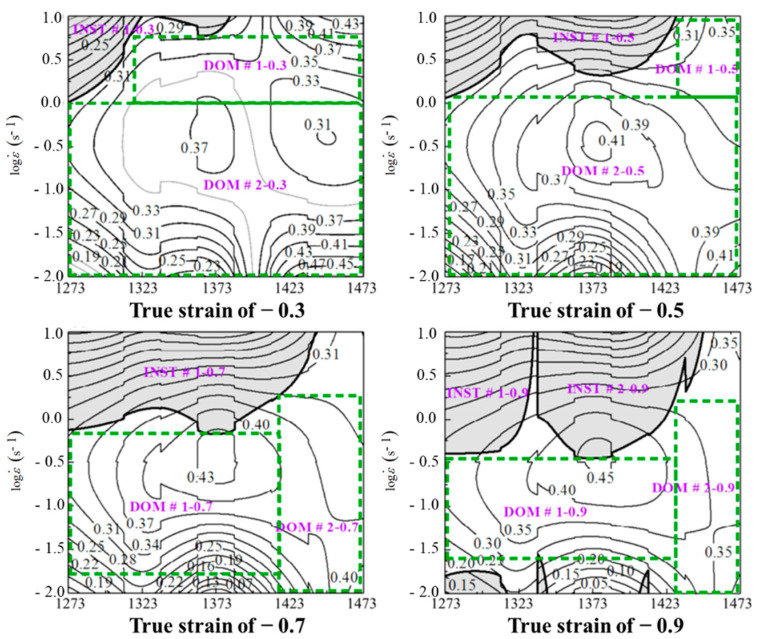
Schematic illustration of processing maps at discrete strains.

In recent years, so many studies about describing the microstructural evolution mechanisms of an alloy have been conducted by the theory of processing maps. As for Ni80A superalloy, Quan et al. [14] constructed a superimposed map of the decline ratio of flow stress to describe the relationship of flow stresses, microstructures, and dynamic softening mechanisms. Quan et al. [15] separated the deformation parameter windows with DRX mechanisms in an enhanced processing map and identified the desired parameter domains corresponding to the DRX mechanisms and lower energy barrier. Wen et al. [16] analyzed the relationships between microstructures and deformation parameters by the constructed processing maps of Ni3Nb alloy, and then identified the optimum deformation parameter domains for ingot cogging, conventional die forging, and isothermal die forging, respectively. Łukaszek-Sołek et al. [17] characterized the workability of Ni-Fe-Mo alloy by the complex processing maps and combining both processing parameters and microstructural evolution. In summary, these works identified the parameter domains corresponding to different microstructural evolution mechanisms, but these identification results were difficult to describe the continuous influence caused by strain rate change during deformation. This is due to the fact that a piece of 2D processing map could only indicate the continuous relationships between indicators, strain rate, and temperature under a constant strain. In order to introduce the influence caused by strain rate change during deformation into the description work, a series of 2D processing maps of discrete strains are constructed by most researchers. MOKDAD et al. [18], Quan et al. [19], Jiang et al. [20], and Park et al. [21] constructed the processing maps considering a discrete strain of Al−Cu−Mg nanocomposite, 42CrMo high-strength steel, alloy 617B, and Ti–6Al–4V alloy. Their research methods were similar, and the typical processing maps of Ni80A superalloy were constructed by Quan et al. [15] as a schematic illustration for this method. As shown in Figure 1, the processing maps under four discontinuous strains are plotted. However, the identified parameter domains at discrete strain are always local and discrete. In fact, strain rate is a dynamic and time-variant process parameter and determines the developing of a thermo–plastic deformation process. It means that the above approach is unable to clarify the dynamic evolution of microstructure mechanisms precisely. Furthermore, the above approach does not support the loading path design of press speed, a process variable determined by strain rate.

**Figure 2:**
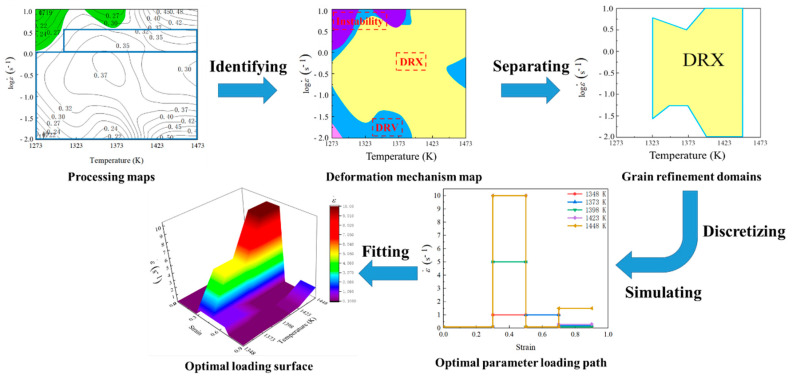
Procedures of parameter loading path design for grain refinement.

**Figure 3:**
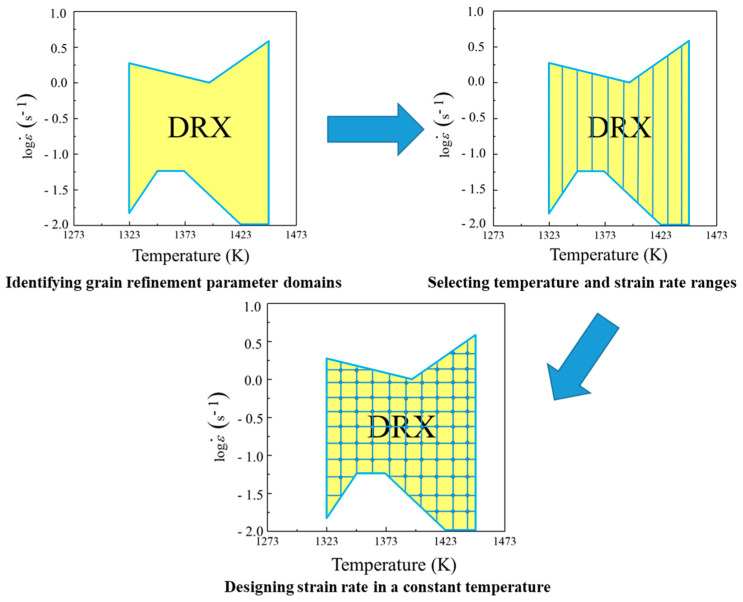
Schematic illustration of grain refinement domain discretization.

**Figure 4:**
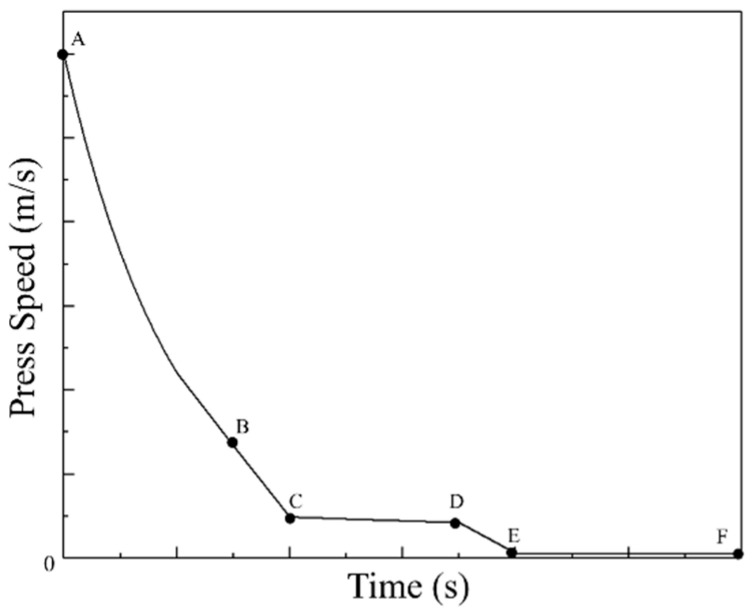
Variation of press speed with time.

**Figure 5:**
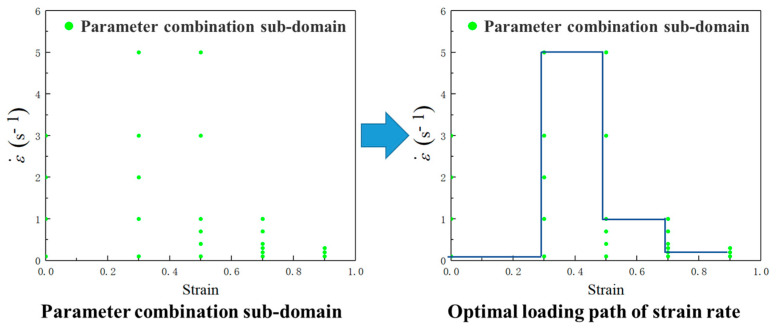
Design of optimal loading path of strain rate.

It is a difficult and significant issue how to establish an approach to identify the parameter domains with desirable microstructural mechanisms at continuous strain rate, strain, and temperature, and then design the loading path of press speed for acquiring the desirable microstructures. Here, an innovative approach of loading parameter design for grain refinement is proposed as Figure 2, and it has been applied in the compression process of Ni80A superalloy. As for such an innovative approach, the detailed procedures of loading parameter design are divided into five steps. The prerequisite of innovative loading parameter design is to identify the parameter domains corresponding to grain refinement from a chaotic system of processing parameters. Firstly, the total deformation strain is divided into four stages according to the strain extending, i.e., 0–0.3, 0.3–0.5, 0.5–0.7, and 0.7–0.9, respectively. Meanwhile, the processing maps corresponding to the strains of 0.3, 0.5, 0.7, and 0.9 are constructed, from which the parameter domains with stable deformation mechanisms are clarified. Secondly, the deformation mechanism maps are constructed by combining the evaluation indicators of processing maps with microstructure observation results. Thirdly, the desirable parameter domains with the stable deformation mechanisms and the grain refinement mechanisms are separated from the chaotic system of processing parameters. Even so, those domains are still sizable chaotic spaces. It is still difficult how to innovatively design the optimal strain rate–loading path from those domains. Therefore, it is a reasonable solution to discretize the grain refinement domains into a limited series of sub-domains that consider temperature and strain rates as variables to make these sizable chaotic spaces clearer. Five temperatures are selected in the temperature range of grain refinement domains, and then the strain rate range of each grain refinement domain at each selected temperature is determined. According to the determined strain rate range, different constant strain rates are designed. That is, each sub-domain can be considered as a specific combination of strain, temperature, and strain rate through the division of total deformation strain and the discretization of grain refinement domain as shown in Figure 3. Fourthly, all the optimal strain rates at all the combinations of temperature and strain are connected as the optimal parameter loading paths of strain rate. It relies on a series of finite element simulations of how to conduct the determination of optimal strain rate. In the actual deformation process, the main adjusting variable is press speed, which can be directly calculated from strain rate [22]. Therefore, the strain rate is determined as the processing parameter to be optimized. An illustration example about the change of press speed over time under varying strain rate is shown in Figure 4, in which the strain rates of sections AB, CD and EF are constant. The average grain size is used as the evaluation indicator, and its value of each parameter combination sub-domain is acquired through step-by-step finite element simulation. In each deformation stage, the smaller the average grain size is, the better the strain rate. The optimal loading path of strain rate is formed by connecting the optimal-parameter combination sub-domains in each deformation stage, as shown in Figure 5. Finally, the optimal loading path surface of strain rate as a function of temperature and strain is fitted by interpolating the optimal- parameter loading paths.

## 2. Basis Theory of Deformation Mechanism Map

In the proposed innovative method, the most critical role is to construct the deformation mechanism map. In 1983, Dynamic Materials Model (DMM) theory was proposed by Prasad et al. [23], and based on this, the deformation mechanism map was constructed and employed to illustrate the relationships of deformation mechanisms, temperatures, strain rates, and strains. In DMM theory, the thermo-plastic deformation is regarded as an energy dissipation process, which includes plastic deformation dissipation (*G*) and microstructural evolution dissipation (*J*). The ratios of *G* and *J* are determined by the strain rate sensitive index (*m*) under specific conditions, and the *m*-value can be calculated by Equation (1) [24]. For steady-state flow stress, the *m*-value varies from 0 to 1, indicating different energy dissipation states, which can be expressed by the power dissipation efficiency(*η*) in Equation (2). Different *η*-values imply different deformation mechanisms, containing DRX, DRV, and cracking. Usually, the larger *η*-values correspond to the softening mechanisms for refinement microstructure, but it is unequal to the stable deformation mechanisms [25]. Therefore, the stable domain is further identified through the instability criterion as Equation (3), and the domain where instability parameter (*ζ*) is greater than zero represents the stability domain. Based on the above description, the processing maps of alloys can be constructed.

Although processing maps provide an approach to clarify the relationships between microstructure evolution mechanisms and processing parameters, the identification criteria need to be further determined with the help of microstructure observation. By characterizing the microstructures of the isothermally compressed specimen under different deformation conditions, the dominant microstructure evolution mechanism, DRX or DRV, corresponding to those conditions is determined. Then, the relative critical *η*-values corresponding to various microstructure evolution mechanisms are determined accurately. Based on the different critical *η*-values, a three-dimensional response space with different clarified mechanism domains is developed, and it is named as the deformation mechanism map.

(1) $$m=\frac{\partial J}{\partial G}=\frac{\dot{\varepsilon }\partial \sigma }{\sigma \partial \dot{\varepsilon }}=\frac{\partial \mathrm{lg}\sigma }{\partial \mathrm{lg}\dot{\varepsilon }}$$

(2) $$\eta =\frac{J}{J_{\max}}=\frac{2m}{m+1}$$

(3) $$\xi (\dot{\varepsilon })=\frac{\partial \mathrm{lg}\frac{m}{m+1}}{\partial \mathrm{lg}\dot{\varepsilon }}+m<0$$

## 3. Clarification of Grain Refinement Domains

### 3.1. Elaboration of Deformation Mechanisms 

**Figure 6:**
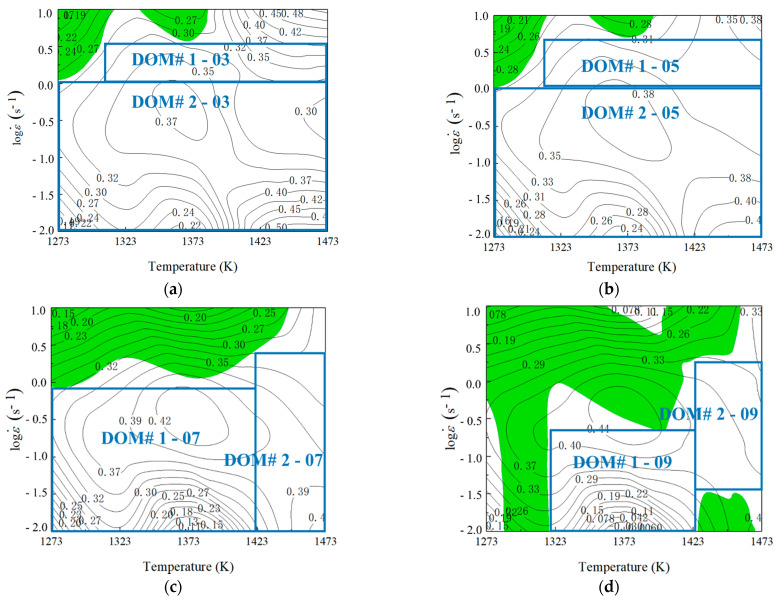
Processing maps at different true strains: (**a**) 0.3, (**b**) 0.5, (**c**) 0.7, and (**d**) 0.9.

In our previous work, Quan et al. [15] constructed the processing maps of Ni80A superalloy under the strains of 0.3, 0.5, 0.7, and 0.9. Additionally, the results are exhibited in Figure 6. In Figure 6, the domain with *ξ*-value greater than 0, and high *η*-value (greater than 0.2) was considered as the stability domain with good workability. In each processing map, the instability domain and the stability domain were denoted by green-shaded domain and white domain, respectively, in which the stability domains with different processing parameters were distinguished by blue lines, followed by the ranges of safety parameters being obtained and explained in Table 1.

Under a true strain of 0.916, the microstructures at different temperatures and strain rates were characterized in details by the work of Quan et al. [15]. The characterized microstructures correspond to DOM#1 and DOM#2 in Figure 6d. The processing parameters of these characterized microstructures were a constant strain rate of 0.1 s^−1^, temperatures of 1273 K, 1323 K, 1373 K, 1423 K, and constant temperature of 1473 K, and strain rates of 0.01 s^−1^ and 0.1 s^−1^, respectively. Based on the obtained characterization results of the microstructures at different processing parameters, the relative critical *η*-values corresponding to various microstructure evolution mechanisms were identified. The microstructures under a strain rate of 0.1 s^−1^ and temperature of 1273 K showed that some DRX grains could be observed at the original grain boundary, which was a representative characteristic of DRV. Under this deformation condition, DRV was dominant. Observed next were the microstructures under a strain rate of 0.1 s^−1^, temperatures of 1323 K, 1373 K, and 1473 K, and the deformation temperature of 1473 K, and strain rates of 0.01 s^−1^ and 0.1 s^−1^. It was found that more DRX grains could be found and uniformly distributed along the original grain boundary, suggesting that DRX had occurred completely and played a dominant role. Contrasting these microstructures with the corresponding domains in processing map, it was found that DRX occurred when *η*-values were greater than 0.3, and DRV occurred when *η*-values were less than 0.3. That is to say, the critical *η*-value of DRX was 0.3.

**Figure 7:**
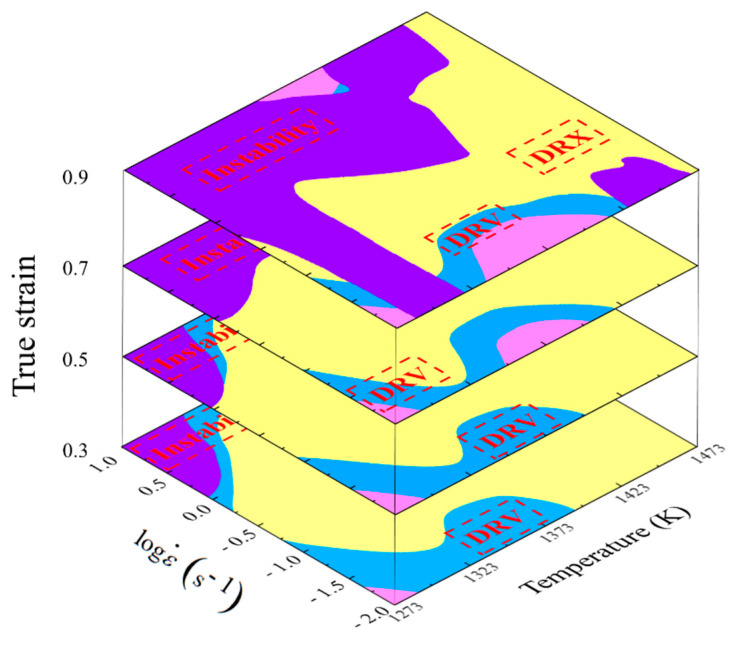
Deformation mechanism map of Ni80A superalloy.

Based on the determined critical *η*-values of DRX in the processing maps, the domains with *η*-values greater than 0.3 were identified as DRX domain, and the domains with *η*-values less than 0.3 were identified as DRV domain. With that, the processing parameter domains corresponding to DRX, DRV, and instability mechanisms were clarified, following which, the deformation mechanism maps of Ni80A superalloy were constructed as shown in Figure 7. In these maps, the clarified domains of DRX, DRV, and instability mechanisms were marked with yellow, purple, and blue, respectively.

### 3.2. Separation and Discretization of Grain Refinement Domains

**Figure 8:**
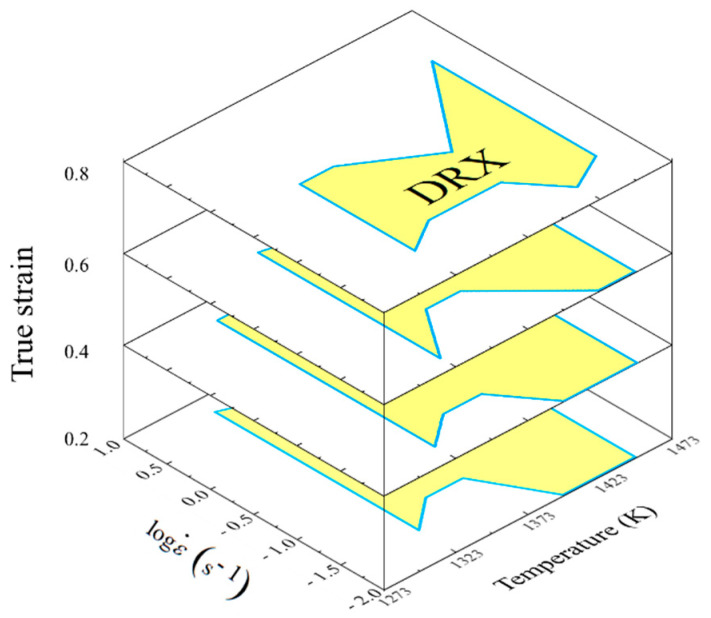
Grain refinement domains for Ni80A superalloy.

**Figure 9:**
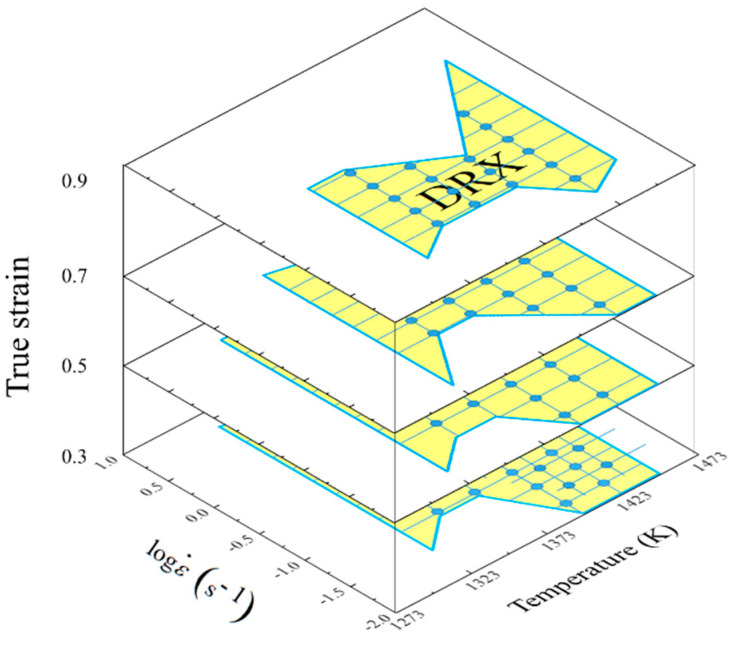
Discretization of grain refinement domains.

It is well accepted that grain size evolution was determined by the game relation of DRX and grain coarsening in thermo-plastic deformation. Since the stable parameter domains of DRX had been clarified, it is valuable to separate and discretize the grain refinement domains aiming to realize the innovative method of parameter loading path design. Therefore, the desirable parameter domains with stability deformation mechanisms and DRX mechanisms were separated from the deformation mechanism map, as shown in Figure 8, wherein the yellow domain represented grain refinement domains. It can be summarized from Figure 8 that the temperature range of grain refinement domains was 1323–1448 K. In addition, features highlighting the domains with deformation temperature, strain, and strain rate were irregular. In other words, the separated grain refinement domain was still too chaotic to design the optimal loading path. Therefore, for an isothermal compression process, the total deformation strain was divided into four stages according to the strain extending: I—the strain range of 0–0.3, II—the strain range of 0.3–0.5, III—the strain range of 0.5–0.7, and IV—the strain range of 0.7–0.9. Taking temperature and strain rate as two variables, the grain refinement domains were discretized into a finite series of sub-domains with clear processing parameters as shown in Figure 9. Such discretization made those domains more clear. With that, for a specific temperature, the strain rate ranges of grain refinement domain at different stages were identified to make preparations for determining the optimal loading path, as listed in Table 2.

## 4. Design in Optimal Parameter–Loading paths

### 4.1. Design Procedures of Optimal Parameter–Loading Path

**Figure 10:**
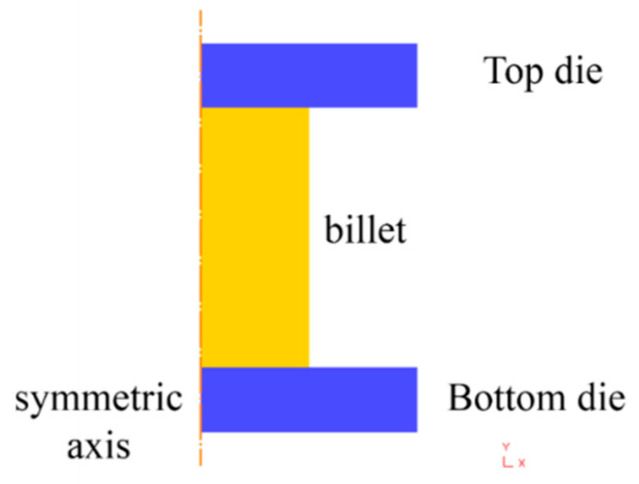
Finite element model for isothermal compression process.

As mentioned above, for the isothermal compression process of Ni80A superalloy, the optimal parameter loading path of strain rate was designed by a finite element method in DEFORM-2D simulation software. The finite element model was established as shown in Figure 10. In this model, the billet was simplified as a cylindrical specimen with a diameter of 10 mm and a height of 12 mm. The billet was set as a rigid-plastic body, and the two anvils were defined as rigid bodies. Based on quadrilateral mesh method, each anvil was meshed as 1110 elements, and billet was meshed as 5172 elements. The heat transfer coefficient between the billet and two anvils was defined as 0.033. The friction coefficient was assumed as a shear type and taken as 0.3. The pressing speed was applied to top die, and it can be calculated based on strain rate as Equation (4) [22].

(4) $$v=h_{0}\dot{\varepsilon }\exp\left(-\dot{\varepsilon t}\right)$$

The DRX kinetic model and grain growth model of Ni80A superalloy constructed by Quan et al. [13] were embedded into DEFORM-2D software to uncover the grain size evolution, and their expressions were as follows [13]:

(5) $$\left\{ \begin{array}{c} X_{\text{DRX}}=1-\text{exp}\left[-1.126{\left(\frac{\varepsilon -\varepsilon_{c}}{\varepsilon_{0.5}}\right)}^{2.183}\right]\\ \varepsilon_{0.5}=0.007946{\dot{\varepsilon }}^{0.06175}\text{exp}(\frac{44,241}{RT}) \end{array}\right.$$

(6) $$d_{\text{drx}}=2380.8{\dot{\varepsilon }}^{-0.0992}\text{exp}(\frac{-63,249}{RT})$$

The initial grain size was set as 34.8 μm. In addition, the relevant parameters of Ni80A superalloy, Young’s modulus, and thermal conductivity, were from the material parameters in Deform material library, as shown in Table 3 and Table 4, respectively. 

The specific design procedures of optimal parameter loading path at a fixed temperature were as follows: Firstly, the average grain size at different strain rates in stage I was obtained, and the optimal strain rate of stage I was determined by comparing the average grain size. Secondly, based on the optimal strain rate of stage I, the simulation of stage II was carried out, and the optimal strain rate of stage II was obtained in the same way. The optimal strain rates at all four stages are determined in the same manner, respectively. Finally, by connecting the optimal strain rate in each deformation stage, the optimal parameter loading path of strain rate at this deformation temperature was obtained.

### 4.2. Descriptions of Optimal Parameter Loading Path 

**Figure 11:**
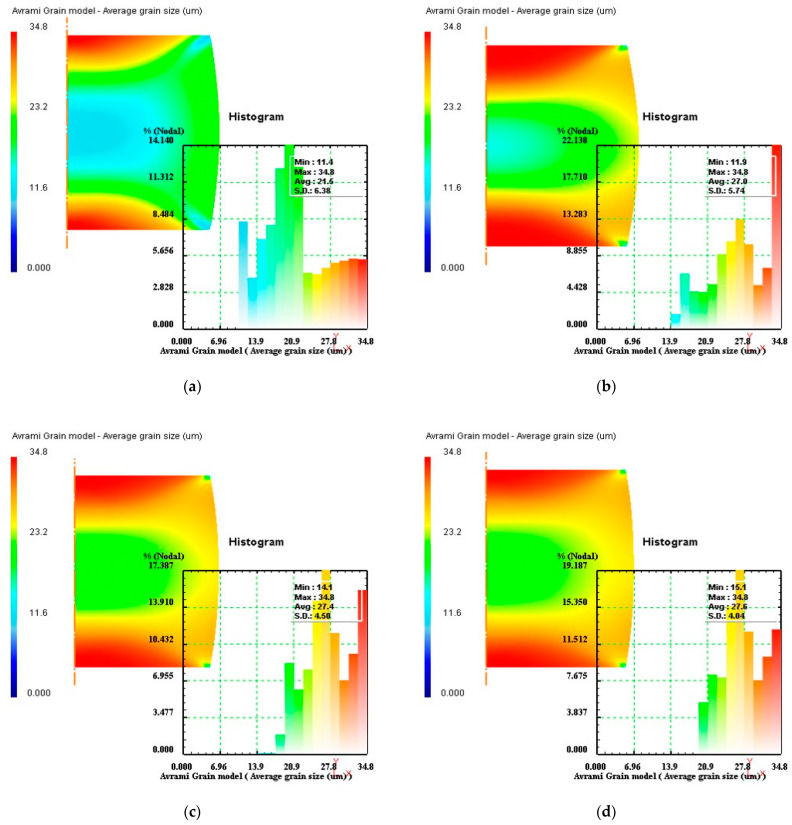
Average grain size distributions of stage I under different strain rates: (**a**) 0.1 s^−1^, (**b**) 3 s^−1^, (**c**) 7 s^−1^, and (**d**) 10 s^−1^.

According to the design procedures of optimal parameter loading path, the simulations of the isothermal compression processes of Ni80A superalloy under the processing parameters in Table 2 were performed. Taking the specimen deformed under the temperature of 1423 K as an example, the average grain size distributions of stage I under different strain rates of 0.1 s^−1^, 3 s^−1^, 7 s^−1^, and 10 s^−1^ were demonstrated as Figure 11. In Figure 11, the values of average grain size were measured as 21.5 µm, 27.0 µm, 27.4 µm, and 27.6 µm, respectively. The comparisons showed that at the strain rate of 0.1 s^−1^, the average grain size was conspicuously smaller in comparison to other strain rates. Therefore, the optimal strain rate in stage I was determined as 0.1 s^−1^.

**Figure 12:**
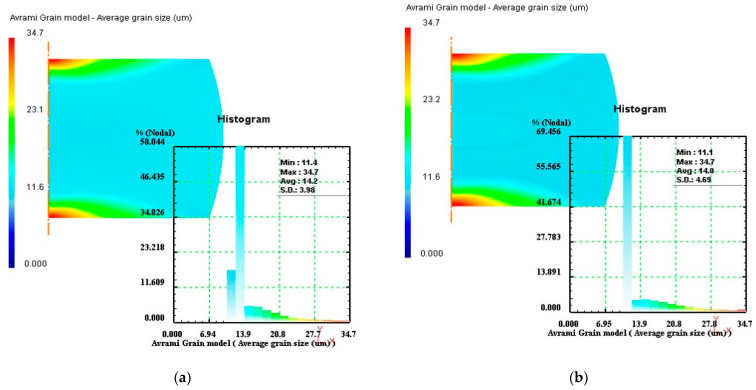

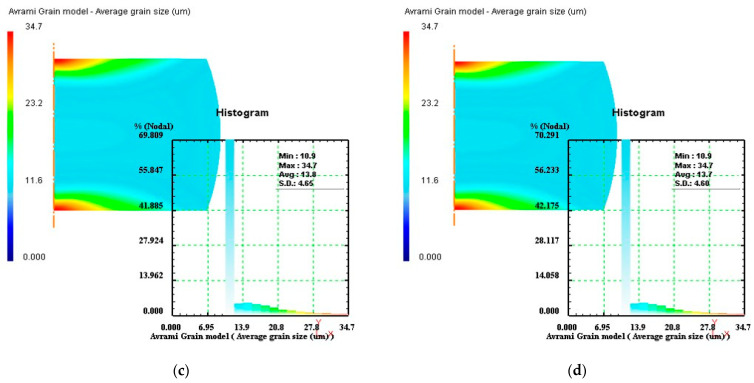
Average grain size distributions of stage II under different strain rates: (**a**) 0.1 s^−1^, (**b**) 3 s^−1^, (**c**) 7 s^−1^, and (**d**) 10 s^−1^.

**Figure 13:**
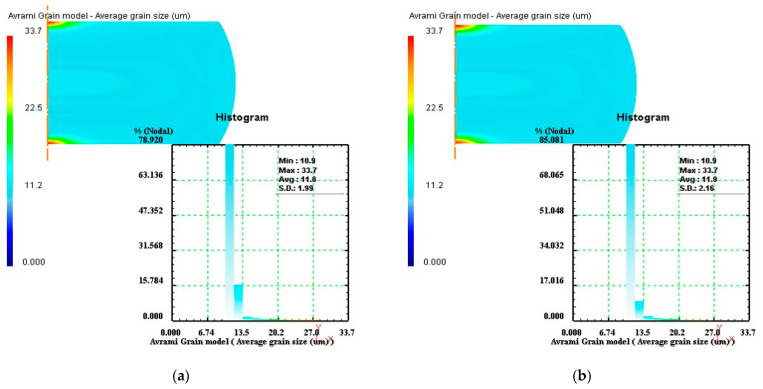

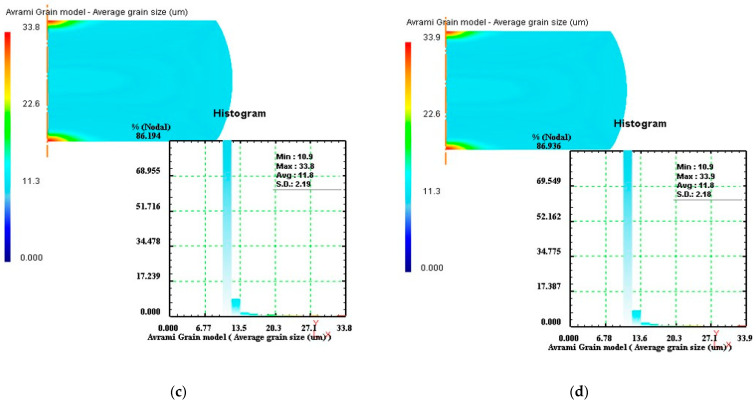
Average grain size distributions of stage III under different strain rates: (**a**) 0.1 s^−1^, (**b**) 0.5 s^−1^, (**c**) 1 s^−1^, and (**d**) 1.4 s^−1^.

**Figure 14:**
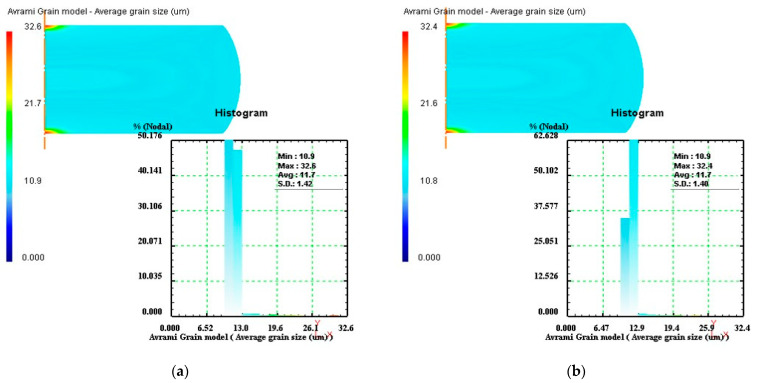

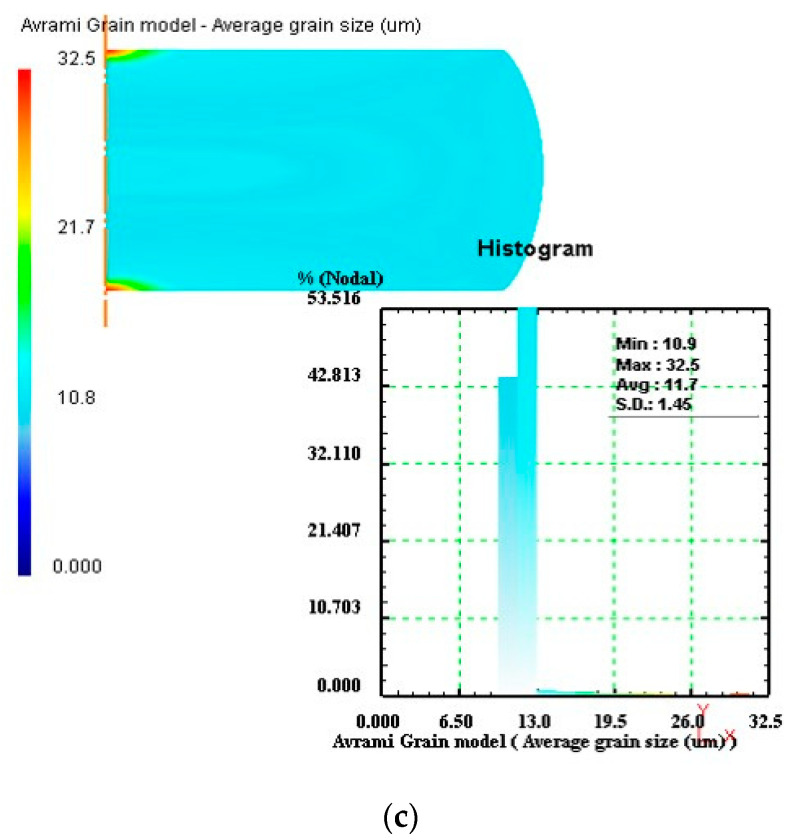
Average grain size distributions of stage IV under different strain rates: (**a**) 0.1 s^−1^, (**b**) 0.3 s^−1^, and (**c**) 0.5 s^−1^.

Based on the optimal strain rate of stage I (0.1 s^−1^), the simulation of stage II was performed at different strain rates, and the strain rate corresponding to finer average grain size was regarded as the optimal strain rate in stage II. The average grain size distributions of stage II under different strain rates of 0.1s^−1^, 3 s^−1^, 7 s^−1^, and 10 s^−1^ were exhibited in Figure 12. In Figure 12, the values of average grain size were measured as 14.2 µm, 14.0 µm, 13.8 µm, and 13.7 µm, respectively. The results indicated that the optimal strain rate in stage II was 10 s^−1^. Similarly, the optimal strain rates of stage III and stage IV would be designed, and their corresponding simulation results were shown in Figure 13 and Figure 14, respectively. By comparing the average grain size and its standard deviation at these strain rates, it was obtained that the optimal strain rate of stage III was 0.1 s^−1^, and the optimal strain rate of stage IV was 0.3 s^−1^.

By connecting the optimal strain rate at those stages, the optimal loading path of strain rate was obtained. Similarly, the optimal loading paths of strain rate at the temperatures of 1348 K, 1373 K, 1398 K, and 1448 K also were designed, and the obtained strain rate at stages of I–IV was given in Table 5. 

### 4.3. Fitting Optimal Parameter Loading Path

**Figure 15:**
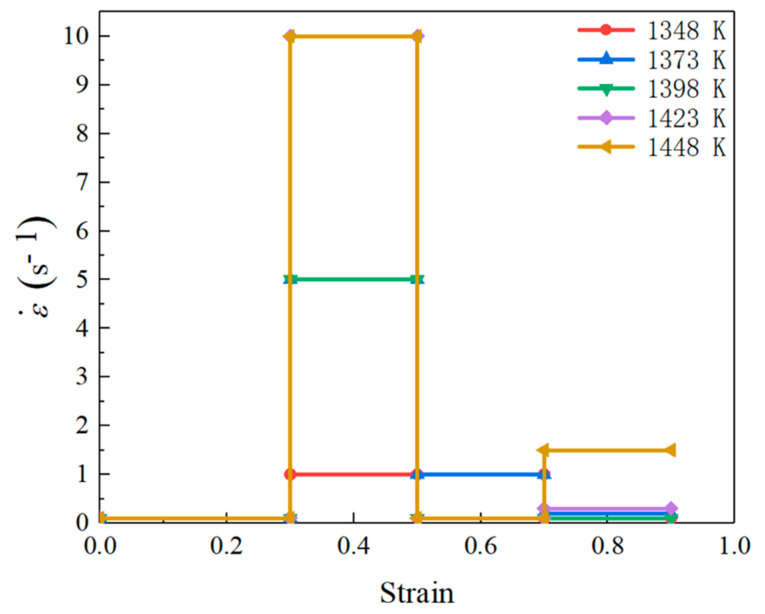
Optimal loading paths of strain rate at different temperatures.

**Figure 16:**
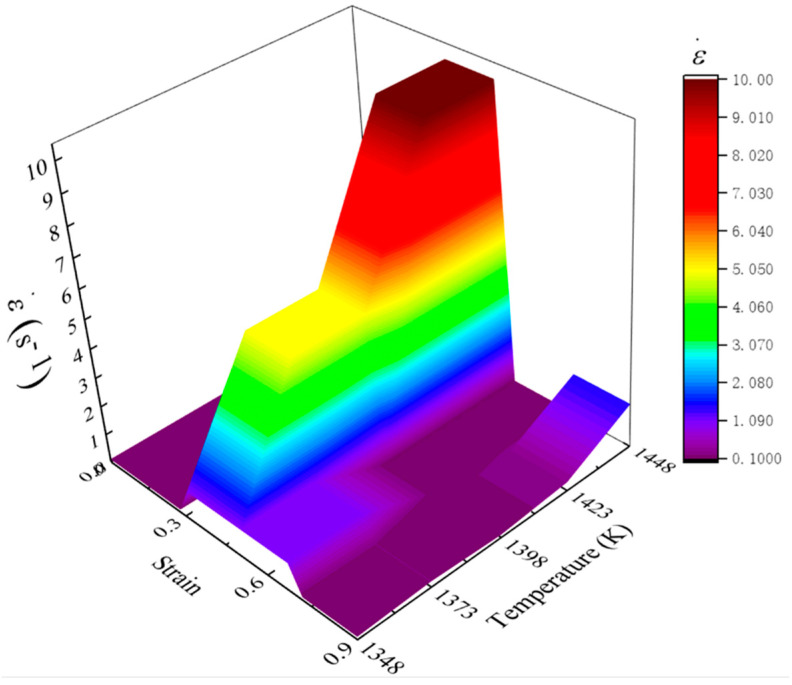
A three-dimensional response surface of the optimal loading path of strain rate as a function of temperature and strain.

According to the designed optimal strain rates in Table 5, the optimal loading path of strain rate at the studied temperatures were drawn and shown in Figure 15. In all deformation temperatures, a common feature of these loading paths was that the grain can be refined effectively at a lower strain rate for stage I. In stage II, the optimal strain rate increased dramatically, and the optimal strain rate decreased with the decreasing of temperature. In the stages of III–IV, the magnitude of optimal strain rate decreased to a lower level again. According to Figure 15, a 3D response surface of the optimal loading path of strain rate as a function of temperature and strain was fitted by interpolating the method, as shown in Figure 16. Such optimal parameter loading path surface can be utilized to guide actual process design of Ni80A superalloy, aiming to obtain the desired mechanical properties of products.

### 4.4. Verifications of Optimal Parameter Loading Path

**Figure 17:**
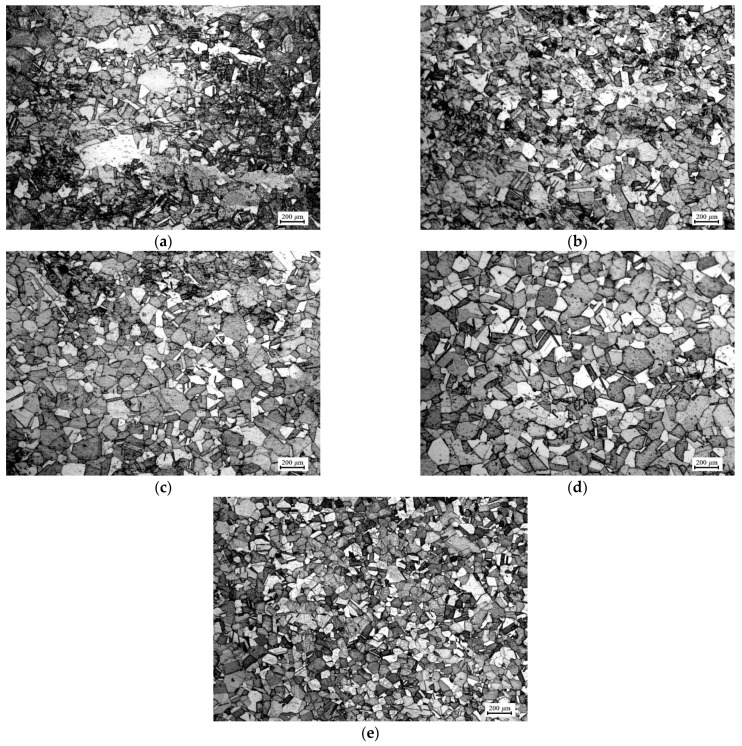
Optical microstructures with optimal loading paths of strain rate under different temperatures: (**a**) 1348 K, (**b**) 1373 K, (**c**) 1398K, (**d**) 1423 K, and (**e**) 1448 K.

The physical experiments of isothermal compression for Ni80A superalloy were carried out on a Gleeble-3500 simulator. Based on the obtained optimal loading paths of strain rate in Table 5, the specimens were compressed to a true strain of 0.916 under the temperatures of 1348 K, 1373 K, 1398 K, 1423 K, and 1448 K, respectively. Similarly, the pressing speed was calculated based on the optimal loading path of strain rate in Table 5 according to Equation (4). After compression, the microstructures of these specimens were characterized using optical microscope technology. More of the experiment’s details were represented than in our previous work [14]. Figure 17 exhibits the microstructures under these compression conditions. It was observed from Figure 17 that the finer grains were distributed uniformly in the microstructures, which indicated that the obtained optimal parameter–loading path could refine grains effectively. It was confirmed that the innovative method of parameter loading path design in the separated grain refinement domain was beneficial and practical to grain refinement.

## 5. Conclusions

An innovative approach of loading parameter designs for grain refinement has been proposed, which was applied to the compression process of Ni80A superalloy. The main conclusions of this work are as follows:

(1) The deformation mechanism map derived from processing maps was constructed, since the critical *η*-values corresponding to DRX and DRV mechanisms were determined. The processing-parameter domains with DRX mechanism were clarified and separated.

(2) A finite series of sub-domains with clear processing parameters were discretized from the separated grain refinement domains, and the optimal strain rate of each sub-domain was determined by simulation. The obtained optimal loading paths of strain rate for four strain stages of 0–0.3, 0.3–0.5, 0.5–0.7, and 0.7–0.9 were as follows: 0.1–1–1–0.1 s^−1^ and 1348 K, 0.1–5–1–0.2 s^−1^ and 1373 K, 0.1–5–0.1–0.1 s^−1^ and 1398 K, 0.1–10–0.1–0.3 s^−1^ and 1423 K, and 0.1–10–0.1–0.5 s^−1^ and 1448 K. These optimal parameter loading paths were finally fitted as a 3D response surface.

(3) The verification experiments of the obtained optimal parameter loading path indicated that the desired grain refinement microstructures of Ni80A superalloy were achieved using the proposed innovative method. This method also can contribute to the microstructure adjustment of other alloys with DRX mechanism.

## Figures and Tables

**Table 1:** Safe parameter range at the true strain 0.3, 0.5, 0.7, and 0.9.

True Strain	Domain	Parameter Range		
		Temperature (K)	Strain Rate (s^−1^)	Peak *η*-Value
0.3	DOM#1–0.2	1300–1473	1.00–3.55	0.37
	DOM#2–0.2	1273–1473	0.01–1.00	0.50
0.5	DOM#1–0.4	1298–1473	1.00–5.01	0.38
	DOM#2–0.4	1273–1473	0.01–1.00	0.42
0.7	DOM#1–0.6	1273–1430	0.01–0.79	0.42
	DOM#2–0.6	1430–1473	0.01–3.16	0.40
0.9	DOM#1–0.8	1310–1415	0.01–0.25	0.44
	DOM#2–0.8	1415–1473	0.04–0.79	0.40

**Table 2:** Range of strain rate at different stages and temperatures.

Stage	Temperature (K)				
	1348	1373	1398	1423	1448
I	0.06–4.47	0.06–3.16	0.01–10.00	0.01–10.00	0.01–10.00
II	0.03–5.62	0.03–5.62	0.01–5.62	0.01–10.00	0.01–10.00
III	0.07–1.41	0.07–1.20	0.02–1.00	0.01–1.58	0.01–3.16
IV	0.07–0.56	0.07–0.35	0.07–0.18	0.02–0.50	0.03–1.78

**Table 3:** Thermal conduction of Ni80A superalloy.

T/°C	100	200	300	400	500	600	700	800	900
k/(N/s)	12.11	13.83	15.48	16.75	18.39	20.93	24.48	25.57	27.76

**Table 4:** Young’s modulus of Ni80A superalloy.

T/°C	20	200	400	600	800	900	1000
E/Pa	219,000	210,000	197,000	183,000	165,000	153,000	141,000

**Table 5:** Optimal strain rate at different stage and temperature of Ni80A superalloy.

Stage	Temperature (K)				
	1348	1373	1398	1423	1448
I	0.1	0.1	0.1	0.1	0.1
II	1	5	5	10	10
III	1	1	0.1	0.1	0.1
IV	0.1	0.2	0.1	0.3	1.5

## Data Availability

The data presented in this study are available on request from the corresponding author.
